# Osteopathic students and graduates matching into pathology residency, 2011–2020

**DOI:** 10.1515/jom-2020-0134

**Published:** 2021-02-01

**Authors:** Ryan Philip Jajosky, Hannah C. Coulson, Abric J. Rosengrant, Audrey N. Jajosky, Philip G. Jajosky

**Affiliations:** Department of Pathology, Laboratory Medicine, Emory University, 201 Dowman Drive, Atlanta, 30322, Georgia, USA; Department of Biomedical Sciences, Discipline of Pathology, Edward Via College of Osteopathic Medicine – Carolinas, Spartanburg, South Carolina, USA; Department of Laboratory Medicine, Geisinger Medical Center, Danville, Pennsylvania, USA; Department of Pathology, Case Western Reserve University, University Hospitals Cleveland Medical Center, Cleveland, Ohio, USA; The Centers for Disease Control and Prevention, Atlanta, Georgia, USA

**Keywords:** education, match, medical students, pathology, recruitment, residency, resident

## Abstract

**Context:**

In the past decade, two changes have affected the pathology residency match. First, the American Osteopathic Association (AOA) Match, which did not offer pathology residency, became accredited under a single graduate medical education (GME) system with the Main Residency Match (MRM), which offers pathology residency. Second, substantially fewer United States senior-year allopathic medical students (US MD seniors) matched into pathology residency.

**Objective:**

To determine whether there were major changes in the number and percentage of osteopathic students and physicians (DOs) matching into pathology residency programs over the past decade.

**Methods:**

Pathology match outcomes for DOs from 2011 to 2020 were obtained by reviewing AOA Match data from the National Matching Services and MRM data from the National Resident Matching Program (NRMP). The number of DOs that filled pathology positions in the MRM was divided by the total number of pathology positions filled in the MRM to calculate the percentage of pathology positions taken by DOs.

**Results:**

Over the past decade, there was a 109% increase in the total number of DOs matching into pathology residency (34 in 2011 vs. 71 in 2020). During this time, there was a 23.3% increase in the total number of pathology positions filled in the MRM (476 in 2011 vs. 587 in 2020). Thus, the percentage of pathology residency positions filled by DOs increased from 7.1% in 2011 to 12.1% in 2020. The substantial increase of DOs in pathology occurred simultaneously with a 94.2% increase in the total number of DOs filling AOA/MRM “postgraduate year 1” (PGY-1) positions (3201 in 2011 vs. 6215 in 2020). Thus, the percentage of DOs choosing pathology residency has remained steady (1.06% in 2011 and 1.14% in 2020). In 2020, pathology had the third lowest percentage of filled PGY-1 residency positions taken by DOs, out of 15 major medical specialties.

**Conclusion:**

The proportion of DOs choosing pathology residency was stable from 2011 to 2020 despite the move to a single GME accreditation system and the stark decline in US MD seniors choosing pathology. In 2020, a slightly higher percentage of DOs (1.14%) chose pathology residency than US MD seniors (1.13%). Overall, DOs more often choose other medical specialties, including primary care. Additional studies are needed to determine why fewer US MD seniors, but not fewer DOs, are choosing pathology residency.

While osteopathic physicians (DOs) and allopathic physicians (MDs) share many similarities in their basic science and clinical training, they also benefit from unique experiences. To become a DO in the United States (US), a student must graduate from an accredited osteopathic medical school. Osteopathic education classically emphasizes the body as a whole (including mind and spirit), hands-on physical examination, the importance of structure and function, disease prevention, and the body’s inherent self-healing capabilities. In contrast, allopathic education often focuses more on symptoms, diagnosis, and specific organ systems. Both allopathic and osteopathic schools teach pathology throughout the first two preclinical years of the curriculum. US state licensing programs and hospitals now recognize the degrees as essentially equivalent, granting DO and MD physicians the same opportunities to specialize and practice.

From 2011 to 2020, two changes had the potential to substantially impact the pathology residency match for DOs. Historically, DOs have had two main options to match into residency programs. Some chose the American Osteopathic Association (AOA) Match, which was exclusively for DOs. Others chose the Main Residency Match (MRM), which is the largest US residency match program and available to all US and international medical students and graduates. In 2016, the AOA began the process of creating a single graduate medical education (GME) accreditation system that combined the AOA Match with the MRM; the process was completed in 2020.^1-10^ Unlike the MRM, the AOA Match did not offer pathology residency training.^11-14^ Single GME accreditation made it less risky for DOs to pursue pathology because they would no longer have to forego the AOA Match to pursue pathology in the MRM. The other major change that affected pathology residency programs was a 27.5% decline^15^ in the number of US allopathic senior-year medical students (US MD seniors) filling pathology residency positions between 2008 and 2017. Notably, this was the largest decline for a major medical specialty^15-17^—a sustained downward trend that, potentially, could have influenced how DOs perceived pathology as a career.

No recent study has tracked how DOs match into pathology residency training, making an assessment warranted given the substantial decline among US MD seniors. Both applicant groups train at US medical schools and are likely exposed to similar cultural influences. To test the hypothesis that DOs would show a similar decline in pathology residency matching, we reviewed AOA Match and MRM data from 2011 to 2020.

## Methods

### Match data

AOA Match data from 2011 to 2020 were obtained from National Matching Services.^11^ Of note, the website housing the AOA Match data is no longer given full technical support, so each year was manually changed in the Uniform Resource Locator (URL) to retrieve the appropriate data. MRM data from 2011 to 2020 were obtained from the National Resident Matching Program (NRMP).^16^ The NRMP reviewed this manuscript before granting written approval to republish MRM data from 2011 to 2020.

### Match-data calculations

The number of DOs who filled pathology positions in the MRM was divided by the total number of pathology positions filled in the MRM to calculate the percentage of DO-filled pathology positions. From 2011 to 2019, the MRM reported the total number of DOs, but in 2020, the data were split into US DO seniors (senior-year osteopathic medical students) and US DO graduates (osteopathic medical students who had graduated). Therefore, solely for 2020, we manually calculated the total number of DOs as the sum of US DO seniors and US DO graduates. The Supplemental Offer and Acceptance Program (SOAP),^18^ for those unable to match in the first round of the MRM, could not be analyzed because a complete set of data is not available on the NRMP website.

The percentage of DOs filling “postgraduate year 1” (PGY-1) residency positions in the AOA and MRM match, the latter of which offered pathology residency, was calculated as follows. First, the number of DOs filling MRM pathology positions was obtained. Of note, pathology only offered PGY-1 positions in the MRM. Then, the total number of DOs filling MRM PGY-1 residency positions was obtained. The number of DOs filling PGY-1 positions in the AOA Match was defined as those filling traditional rotating internships as well as Option 1 and Option 2 residencies. A traditional rotating internship is one year of training that is unaffiliated with a specific medical specialty. An Option 1 residency (e.g., family medicine in the 2017 AOA Match) does not require an internship year. An Option 2 residency (e.g., diagnostic radiology in the 2017 AOA Match) has a preliminary internship year included in the training. These three groups in the AOA Match were considered to represent PGY-1 positions.^19,20^ Option 3 residencies (e.g., dermatology in the 2017 AOA Match) first require a separate internship year not linked to a specific residency. Thus, Option 3 residencies are not PGY-1 positions.^19,20^ PGY-1 positions filled by DOs in the MRM and AOA match were added to determine the total number of DOs filling PGY-1 positions. Finally, the number of DOs filling MRM pathology positions was divided by the number of DOs filling PGY-1 positions in the MRM and AOA Match. These calculations were undertaken to avoid “double counting” individuals, as one person cannot match into multiple PGY-1 positions.

To calculate the percentage of filled residency positions taken by DOs in year 2020, MRM data alone were used, as there was no AOA Match in 2020. Fifteen major medical specialties defined in a prior study were analyzed, including anesthesiology, dermatology, emergency medicine, family medicine, internal medicine, neurology, obstetrics-gynecology, orthopedic surgery, otolaryngology, pathology, pediatrics, physical medicine and rehabilitation, psychiatry, radiology-diagnostic, and surgery.^15^ The numbers of DOs filling the PGY-1 and PGY-2 MRM positions for these 15 specialties were added together. Of note, “physician (R) positions” (which are not available for senior medical students)^16^ were not included. For each specialty, the total number of DO-filled PGY-1 and PGY-2 positions was divided by the total number of PGY-1 and PGY-2 positions filled. Again, these calculations were made with the intention to avoid “double counting” individuals – as one person cannot match into multiple residencies (excluding internship and preliminary years). For example, an MRM applicant can match into “medicine-preliminary” (PGY-1 only) at the same time they match into “radiology (diagnostic)” as a PGY-2 position. According to the 2020 MRM Report, “Applicants can rank multiple specialties. In 2020, 2,336 applicants matched to both PGY-1 and PGY-2 positions; of those applicants, 1,735 were U.S. MD seniors.”^16^

Data were graphed and linear trendlines were added using Microsoft Excel.

## Results

**Figure 1:**
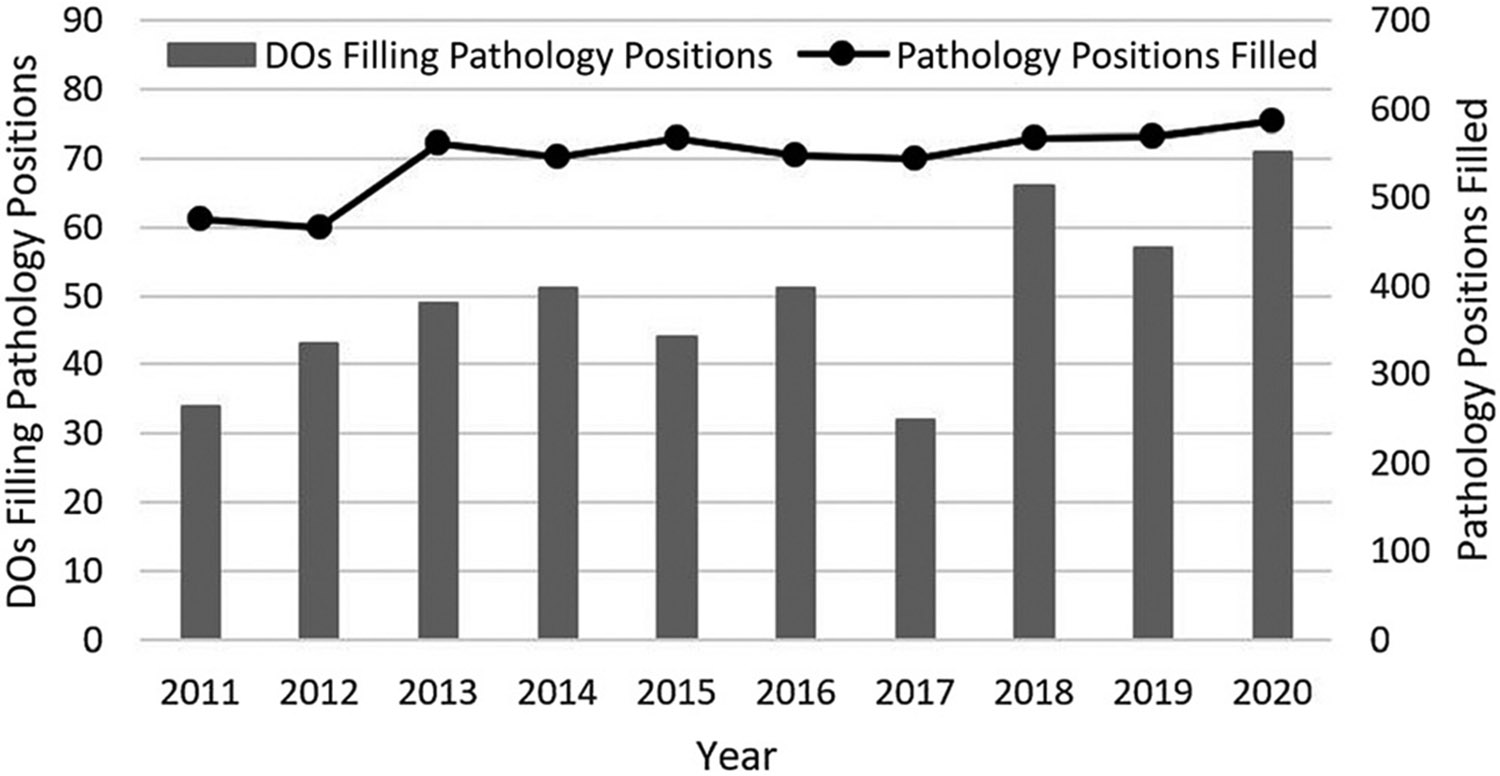
The number of DOs filling pathology residency positions in the MRM, compared to the total number of pathology positions filled, years 2011–2020. MRM, main residency match.

**Figure 2:**
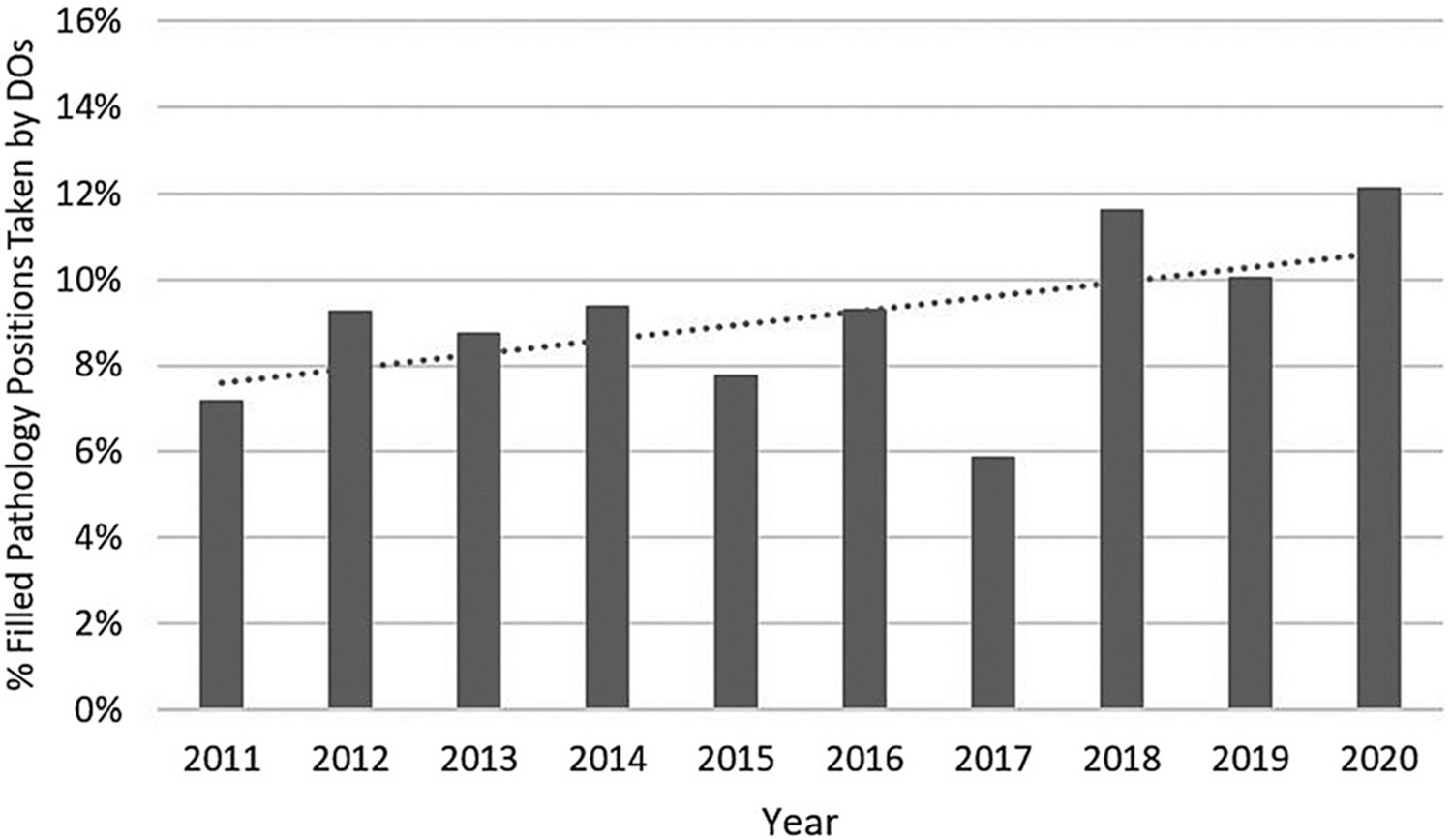
The percentage of pathology residency positions filled in the MRM that were taken by DOs, with a linear trendline. MRM, main residency match.

The number of DOs filling pathology residency positions in the MRM increased by more than double, from 34 in 2011 to 71 in 2020 (109% increase). At the same time, the number of pathology residency positions filled in the MRM increased by only a modest 23.3%, from 476 in 2011 to 587 in 2020 (Figure 1). Thus, the percentage of filled pathology residency positions taken by DOs increased from 7.1% in 2011 to 12.1% in 2020 (Figure 2). Although there was a strong trend of more DOs choosing pathology residency over the past decade, there was a substantial decline in 2017 (an outlier year), followed by a three-year resumption of the long-term rising trend. No obvious trend reversals were noted when comparing the time before single GME accreditation (years 2011–2015) to the time during introduction of single GME (years 2016–2020).

**Figure 3:**
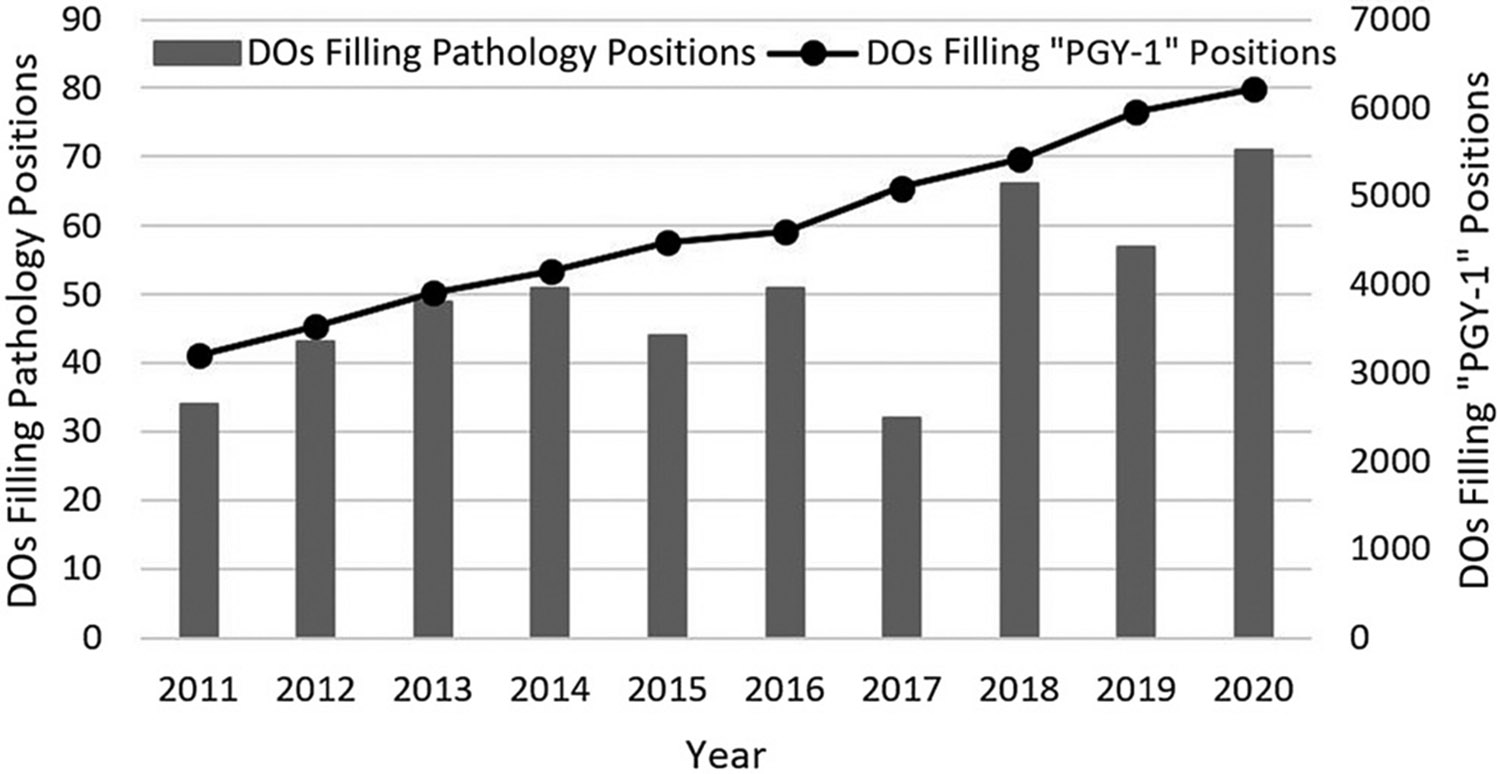
The number of DOs filling pathology residency positions in the MRM, compared with the number of DOs filling “PGY-1” positions. “PGY-1” positions were defined as PGY-1 positions in the MRM and traditional rotating internships and Option 1 and 2 (but not Option 3) residencies in the AOA Match. Option 3 residencies were excluded because, unlike Option 2 residencies, they require a separate internship year. MRM, main residency match.

**Figure 4:**
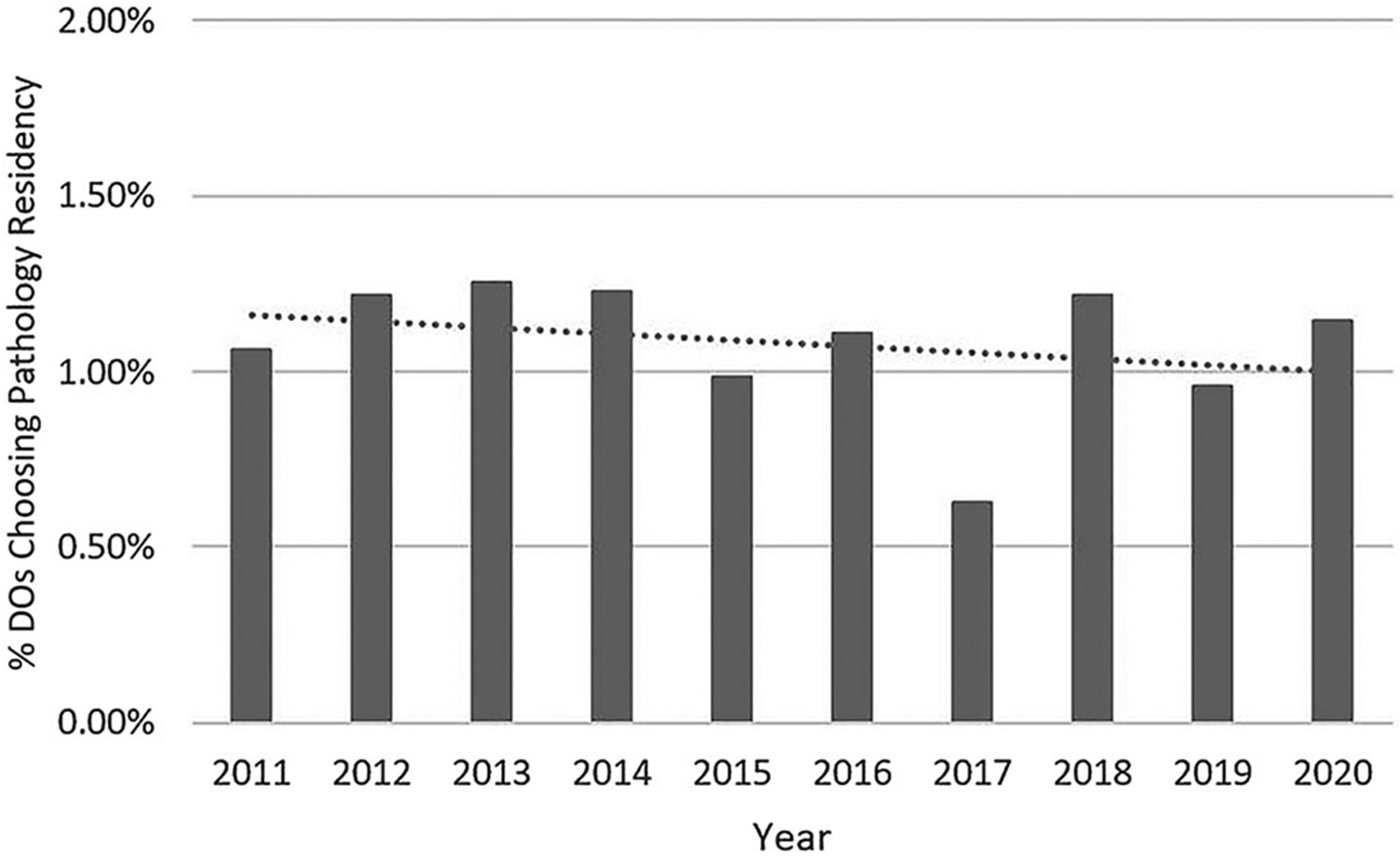
The percentage of DOs matching to a “PGY-1” residency position who chose pathology residency, with a linear trendline.

**Figure 5:**
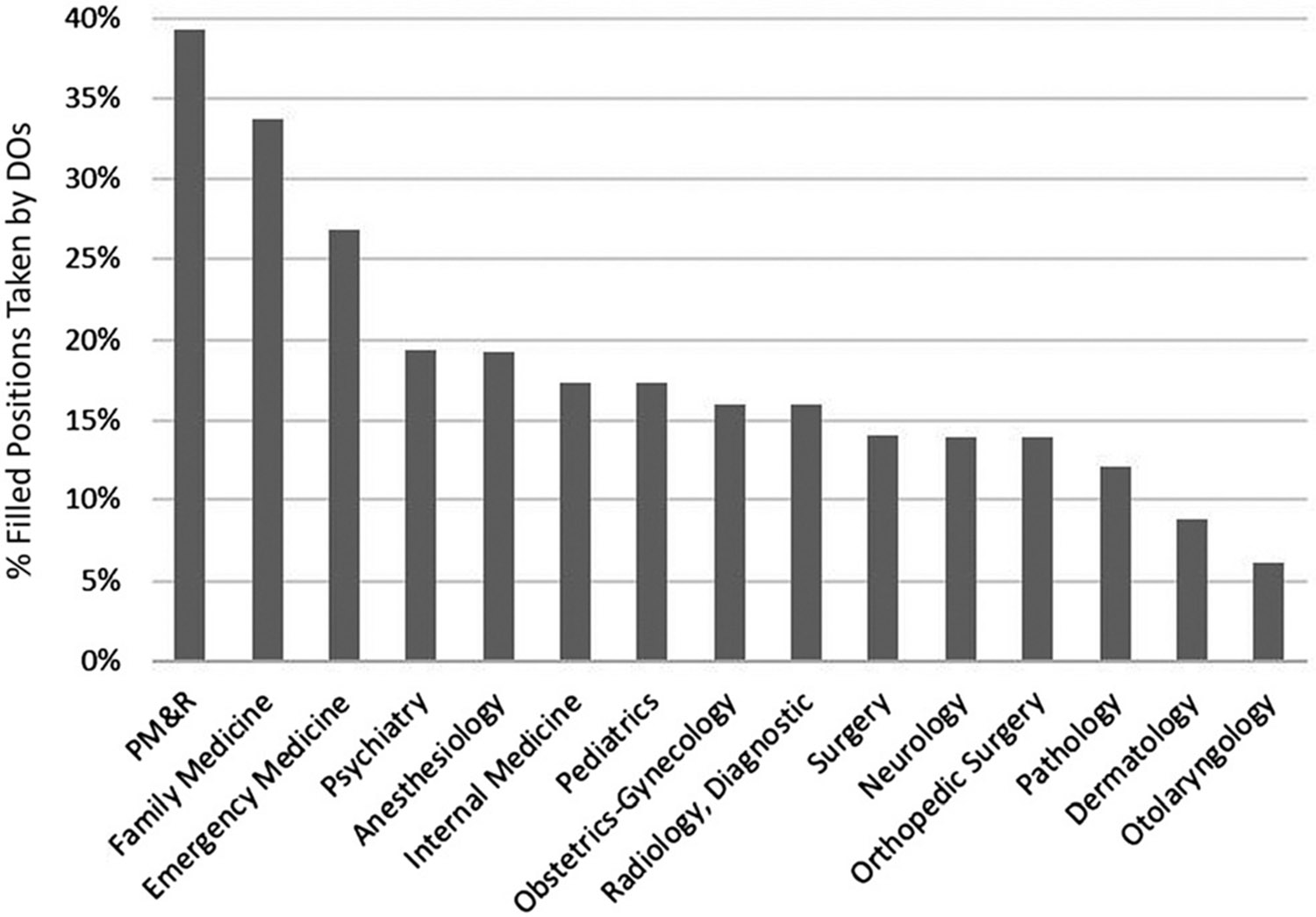
The percentage of residency positions filled in the MRM which were taken by DOs in year 2020. MRM, main residency match; PM&R, physical medicine and rehabilitation.

Next, we studied the proportion of DOs that chose pathology over the past decade. The number of DOs filling PGY-1 positions in the AOA match and MRM almost doubled from 3,201 in 2011 to 6,215 in year 2020 (94.2% increase; Figure 3). This large increase coincides with a significant increase in enrollment at osteopathic medical schools four years earlier. In academic year 2007–2008, first-year enrollment at DO medical schools was 4,528, compared to 7,575 in academic year 2016–2017 (67.3% increase).^21^ It seems that more DOs chose pathology not necessarily due to an increasing interest, but because a greater number of DOs matched into PGY-1 residency positions. Thus, the percentage of DOs choosing pathology increased only slightly from 1.06% in 2011 to 1.14% in 2020 (Figure 4). Overall, the trendline for the entire decade showed a slight decline. Interestingly, no substantial differences were apparent when comparing the time before and during the introduction of single GME accreditation. The percentage of filled pathology residency positions taken by DOs in 2020 (12.1%) was the third lowest of 15 major medical specialties (Figure 5). Among the 15 major medical specialties, a median of 16.0% of residency spots were filled by DOs.

## Discussion

In the past decade, the number of DOs choosing pathology for residency training has more than doubled. Concurrently, nearly twice as many DOs entered PGY-1 residency programs, consistent with the significant growth in osteopathic medical school enrollment.^21^ Thus, the percentage of DOs choosing pathology residency remained relatively stable (at about 1%). Surprisingly, the proportions of DOs matching into pathology before and during introduction of the single GME accreditation system were similar. The low ranking of pathology in terms of percentage of filled residency positions taken by DOs in year 2020 suggests that DOs, perhaps based on the structure of their education, prefer (or are guided toward) other medical specialties.^22^ Interestingly, the combination of single GME and declining interest among US MD seniors^15-17^ did not change the proportion of DOs matching into pathology.

In contrast to DOs, prior reports have shown that both the absolute number and the proportion of US MD seniors choosing pathology have decreased substantially in recent years.^15-17^ From 2011 to 2020, the number of US MD seniors matching into pathology residency decreased from 269 to 204 (24.2% decline), which represented a decrease from 1.73% of US MD seniors choosing pathology to just 1.13% over the decade.^16^ Thus, DOs are not following the trend of US MD seniors.

Our study had several limitations. First, the full impact of single GME accreditation could not be evaluated. This is because year 2020 was the first and only year evaluated in which AOA residency programs were fully integrated into the MRM. A future study will be needed to assess residency trends five years after completion of single CME accreditation system implementation (2021–2025). Another limitation is that the MRM was analyzed, but not the Supplemental Offer and Acceptance Program (SOAP)^18^ for those unable to match in the MRM’s first round. This is because the publicly available SOAP data do not specify the number of DOs or US MD seniors matching into a specific medical specialty.^18^ In addition, pathology residency positions offered outside the MRM were not analyzed.^15,23^ However, the MRM is the largest matching program in the US.^16^ The total number of DOs who applied to pathology residency could not be evaluated because data for the past 10 years were not readily available.^16^ Only 2020 data were available, which showed that 77 US DO senior medical students specified pathology on their Rank Order List (ROL), and 67 filled pathology positions, for an 87% success rate.^16^ Another limitation is that this study did not compare the profiles of osteopathic and allopathic medical students according to gender, ethnicity, age, advanced-degree status, or other demographic markers, nor were differences between osteopathic and allopathic medical school curriculums reviewed. Some schools teach pathology as a distinct course while others use a case-based or systems-based curriculum, where it may not stand-out as a distinct specialty; these educational differences and their influence on eventual residency program matches warrant further study.^24-28^

Although economic factors were not evaluated in this study, specialty salaries warrant attention. Three previous publications^29-31^ in the *Journal of the American Medical Association* have reported strong correlations between the percentage of a specialty’s residency positions filled by US MD seniors and the specialty’s salary. These findings support the idiom that “white follows green,” meaning that physicians (wearing white coats) follow the money (which is green).^32^ However, one of those studies^30^ also included data for DO students and physicians; those results did not show a strong correlation between the percentage of residency positions filled by DOs and specialty salary. Perhaps US MD seniors more strongly consider salary than DOs, and fewer US MD seniors might be choosing pathology due to the relatively lower salaries among pathologists. However, Medscape physician-compensation surveys have shown that pathology salaries ranked 15th out of 25 specialties in the 2012 report^33^ and 16th out of 29 specialties in the 2020 report.^34^ (Of note, pathology salaries were not included in the 2011 report.^35^). Thus, pathology salaries have remained relatively stable compared with other medical specialties. Based on this conflicting data about salary correlation, a more detailed consideration of economic factors is needed.

The decline in US MD seniors choosing pathology has been linked to Student Doctor Network (SDN), a popular social media website for medical students and physicians.^36^ SDN is heavily utilized by both allopathic and osteopathic medical students and might substantially influence residency decisions.^37-41^ On SDN, generally speaking, students will identify a specific forum (e.g., “Medical Students – DO”) and then post a thread (e.g., “AOA Match Results”) where others can comment. A recent study attributed the decline in US MD seniors choosing pathology residency to concerns expressed about the pathology job market on SDN.^42^ Pathology job market concerns on SDN were also described by an allopathic medical student participating in a pathology discussion group.^43^ Furthermore, pathology leadership has also acknowledged this challenge,^44,45^ with the College of American Pathologists (CAP)’s Chair on the Council of Education,^46^ Dr. Donald Karcher, calling it the “studentdoctor.net effect”.^44^

Although DO medical students also use SDN, they do not use the same forum as MD medical students.^47^ As of July 2020, the SDN “Medical Students – DO” forum had 334,900 messages, while the “Medical Students – MD” forum had 814,100 messages.^47^ If the tone of discussion about pathology is different in the MD and DO medical student forums, then this likely reflects differing attitudes about pathology. Thus, we compared the SDN “Medical Student – DO” and “Medical Student – MD” forums. Using SDN’s “search threads” feature, we identified forum threads newer than December 31, 2010 containing the word “pathology” in the thread title. In the “Medical Student – MD” forum, a thread titled “For what reasons do you rule pathology out as a career?” was created in 2016 and has 38 comments.^48^ The commenters describe pathology as “mind-numbingly boring,” “working in a lab,” “low pay,” with “almost zero patient interaction,” associated with “eye strain” from looking into microscopes, with a “chronically horrible job market.”^48^ In the same forum, a more recent thread titled “Interested in pathology. Why is pathology so unpopular? Will it stay unpopular?” was created in 2018 and has 48 comments.^49^ Commenters stated that “path is boring,” “it is highly unlikely that path will get more competitive in the near future,” “the pathology job market is horrible,” and “you don’t [want to] end up in the basement of the ivory tower hating the work you do (pathology).”^49^ Clearly, some allopathic medical students have negative opinions about pathology. In contrast, the “Medical Student – DO” forum did not have such obviously pessimistic threads about pathology. However, a comprehensive study of the pathology threads in these forums would be needed to draw a definite conclusion.

A recent discussion group^43^ with six allopathic medical students revealed other possible reasons why US MD seniors are losing interest in pursuing pathology. The participants felt that many medical students “don’t know what the profession really does,” with one stating, “I was under the impression that all pathologists spent their days performing autopsies in the basement.”^43^ In addition, some students described peer pressure against choosing pathology because the specialty has limited direct patient interaction. One student reported that when they expressed interest in pathology, a classmate responded by saying, “Really? Pathology? That’s kind of weird, isn’t it?”^43^ Another student summarized their experience reading about pathology online, by saying that everything she read said, “Don’t go into pathology.”^43^ Because osteopathic medical students were not part of this discussion group, it is unknown whether they are having similar experiences.^43^ Additional research should be done to analyze the negative perception of this critical specialty and where it originates.

The stable percentage of DOs choosing pathology may be due to multiple factors. In our experience, some DOs had expressed apprehension about forgoing the AOA Match and only participating in the MRM to pursue pathology. Thus, the single GME accreditation system may have increased the relative percentage of DOs pursuing pathology. Concurrently, pathology job market concerns found on SDN may have deterred some DOs from pursuing pathology, just as US MD seniors were deterred. Perhaps competing promoting and deterrent factors negated each other, resulting in a net stable percentage of DOs choosing pathology.

To address the poor recruitment into pathology, CAP announced a new program known as the Pathologist Pipeline Initiative^44,50^ at the CAP’s Spring Resident’s Forum in 2020 and published several papers arguing that the pathology job-market is strong.^51-55^ The Pathologist Pipeline Initiative seeks to improve recruitment of the best medical students, including DOs.^44^ The leader of this initiative, Dr. Donald Karcher, discussed having a “pathology interest group in every US medical school (MD and DO).”^44^ In the future, the pathology-match numbers documented in this study can serve as reference numbers when assessing the effectiveness of these recruitment efforts.^44^ Presently, our findings suggest that DOs and MDs do not seem to appreciate that pathologists are, arguably, the specialists best suited to promote medicine’s cutting-edge frontiers involving assays, cell engineering, and molecular-genetic strategies that will advance patient-specific diagnostics and therapeutics.

## Conclusion

Despite the move to a single GME accreditation system and a significant decline in US MD seniors choosing pathology residency, the proportion of DOs matching into pathology residency has remained relatively stable over the past decade. In the 2020 MRM, a slightly greater percentage of DOs chose pathology than US MD seniors. However, our study did not seek to identify the reasons for the steady stream of DOs into pathology or the reasons that substantially fewer US MD seniors are choosing pathology. The separate trends could be explained by differences in osteopathic and allopathic student demographics, economic factors, or social media influence (e.g., SDN forums), among other things. Future studies, including discussions with DO medical students about pathology perceptions, are warranted to more carefully examine these critical issues. Overall, like US MD seniors, relatively few DOs choose pathology as a medical specialty.
